# An Unusual Case of a Hypopharyngeal Mass in a Patient Presenting With Weakness

**DOI:** 10.1155/crot/5553911

**Published:** 2026-09-04

**Authors:** Christine M. Mavilian, Jun Yun, Alice C. Yu, Chau T. Nguyen

**Affiliations:** ^1^ Department of Head and Neck Surgery, David Geffen School of Medicine at University of California Los Angeles, UCLA, Los Angeles, California, USA, ucla.edu; ^2^ Division of Otolaryngology-Head & Neck Surgery, Ventura County Medical Center, Ventura, California, USA

**Keywords:** hypopharyngeal mass, laryngeal mass, thyroid cancer

## Abstract

In this report, we present the unique case of an incidentally discovered hypopharyngeal mass later found to be papillary thyroid cancer in an 86‐year‐old woman with a past medical history of Parkinson’s disease and papillary thyroid cancer. Though the patient presented with dysphagic and dysphonic symptoms, her history of Parkinson’s disease, presenting symptoms of falls and weakness, and a lack of palpable nodules obscured the diagnosis. Our case emphasizes the importance of maintaining a high clinical index of suspicion for head and neck pathology in patients with a history of thyroid cancer to ensure early recognition of atypical disease presentations.

## 1. Introduction

Hypopharyngeal masses can arise due to benign and malignant etiologies; however, a vast majority of hypopharyngeal masses are malignant [1]. These cancers are uncommon as they comprise less than 5% of all malignant tumors in the head and neck [2]. Histologically, squamous cell carcinomas account for 95% of all cases and are associated with poor prognostic outcomes [1, 3]. Nonspecific symptoms of hypopharyngeal masses include dysphagia, odynophagia, and referred otalgia. Here, we present a unique case of an incidental hypopharyngeal mass detected in an elderly patient with a history of Parkinson’s disease (PD) and papillary thyroid cancer who initially presented with symptoms of weakness and falls.

## 2. Clinical Presentation

**FIGURE 1:**
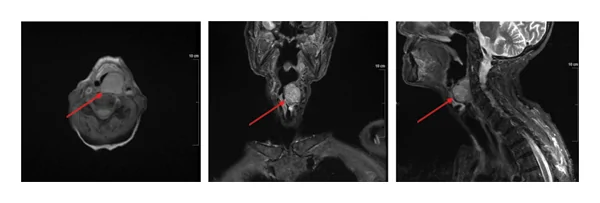
Magnetic resonance imaging revealing a large 3.2 × 2.5 × 3 cm low‐density mass arising in the region of the left posterior supraglottic larynx, some projecting into the supraglottic larynx, narrowing the airway. Red arrows indicate the hypopharyngeal mass.

An 86‐year‐old woman with a past medical history of PD, on Sinemet and Amantadine, and a 3‐cm papillary thyroid cancer, who was previously treated with radioactive iodine, total thyroidectomy in 2011, and radical neck dissection by endocrine surgery in 2019 following lymph node recurrence, was admitted due to weakness and falls. During workup in the emergency department, MRI imaging ordered to rule out stroke incidentally revealed a mass in the hypopharynx, as seen in Figure 1. Upon further questioning, the patient noted progressive hoarseness and dysphagia over the last year that was initially attributed to aging. The patient was discharged for outpatient ENT follow‐up; however, she experienced rapidly progressing weakness in the days following discharge, requiring use of a walker and family support and subsequently re‐presented to the ED for expedited workup and management.

On examination by the otolaryngology team, the patient appeared frail (BMI ∼ 15) and presented with hoarse speech and dry oral mucosa. No neck lymphadenopathy was noted. Auscultation revealed faint crackles at the right lung base with no evidence of wheezing or increased respiratory effort. The patient was afebrile, but laboratory studies were significant for mild leukocytosis, and urine dipstick analysis revealed evidence of blood, proteinuria, and ketones. Radiographic chest imaging was positive for airspace opacities in the right lower lobe that was concerning for an acute infectious process versus aspiration.

**FIGURE 2:**
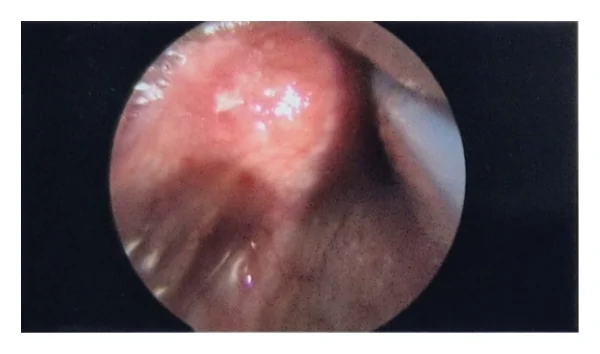
Image from direct laryngoscopy depicting the hypopharyngeal mass causing effacement of the left lateral piriform sinus.

**FIGURE 3:**
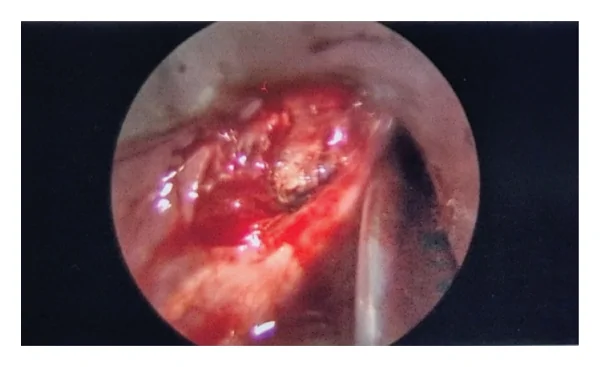
Image from direct laryngoscopy depicting the mass following biopsy.

The patient was diagnosed with community‐acquired pneumonia and was prescribed ceftriaxone and azithromycin. Given the presence of the hypopharyngeal mass, there was concern for aspiration, and the patient was instructed to avoid oral intake for safety. Goals‐of‐care discussions led by the clinicians and speech and language pathologists were conducted with the patient and her son regarding gastrostomy tube (G‐tube) placement for nutritional support and further diagnostic evaluation of the mass versus initiation of hospice care. The patient and her son elected to undergo further workup of the hypopharyngeal mass, but they wanted more time to consider the placement of the G‐tube. The patient underwent direct laryngoscopy with biopsy of the mass and was ultimately found to have metastatic papillary thyroid carcinoma, as seen in Figures 2 and 3. Following this workup, the patient was ultimately lost to follow‐up.

## 3. Discussion

Here, we report a rare presentation of metastatic papillary thyroid cancer in the hypopharynx. Hypopharyngeal masses commonly present with odynophagia, dysphagia, hoarseness, and sore throat, depending on the size of the mass and specific subsite of the hypopharynx involved. In more advanced disease, hypopharyngeal masses that invade the larynx may cause airway obstruction and, potentially, airway compromise [4, 5]. Subacute weakness and falls are an uncommon presentation for a hypopharyngeal mass, contributing to the incidental nature of the discovery of the tumor in this case. Additionally, the discovery that the hypopharyngeal mass represented metastatic papillary thyroid carcinoma was unusual, given that papillary thyroid carcinoma typically favors a lymphatic spread to regional cervical lymph nodes. The most common sites of metastasis of papillary thyroid cancer are the lungs, bones, liver, and brain [6]. However, a few instances of extranodal metastasis to the hypopharynx and adjacent spaces have been documented. For instance, a recent report described hypopharyngeal metastasis of papillary thyroid cancer, which was pathologically confirmed after surgery in a 58‐year‐old patient who presented with 1 month of blood in the sputum [7]. Another report presented the case of a 70‐year‐old woman who developed a rare synchronous involvement of the hypopharynx and parapharyngeal space 10 years after thyroidectomy, with postoperative histopathologic analysis confirming metastatic papillary thyroid cancer [8]. These reports collectively suggest not only that hypopharyngeal metastasis of papillary thyroid carcinoma is uncommon but also that it can occur even years after initial treatment. Therefore, clinicians should maintain a high index of suspicion for recurrence in patients who present with worsening dysphagia and dysphonia with a history of papillary thyroid cancer. Even when dysphagia or dysphonia is presumed secondary to other etiologies, as it was in this case, comprehensive workup remains warranted.

Interestingly, our patient’s history of PD was another factor which complicated her clinical presentation. The patient was diagnosed with PD in 2016, and her symptoms were managed with low dose Sinemet and Symmetrel. According to recent literature, patients with head and neck cancer may have an increased likelihood of subsequently developing PD compared with the general population, although the relationship is associative rather than causal, and there is no evidence that PD predisposes individuals to head and neck cancer [9]. In its advanced stages, PD is characterized by axial motor deterioration, including bradykinesia and postural instability, which are typically generalized and progressive [10]. The neurodegenerative changes in PD also involve the bulbar musculature, leading to dysphagia, dysarthria, and weakness of the pharyngeal muscles as evidenced by prior histopathological and manometric studies [11–14]. While structural changes of the pharyngeal musculature and alpha‐synuclein deposition within pharyngeal tissues have been described in patients with PD [11, 14], the relationship between these neuropathologic changes and the development or progression of hypopharyngeal lesions remains unclear, warranting further investigation into their possible contribution to the patient’s clinical presentation. Given this constellation of findings, the presenting symptoms of hoarseness and dysphagia found in this patient were initially dismissed as part of the natural progression of PD. However, the presence of metastatic papillary thyroid carcinoma in the hypopharynx resulted in an acute deterioration of the patient’s symptoms of weakness shortly before presentation, which contrasts the more progressive decline seen in PD [15, 16]. Thus, a comprehensive evaluation, including neurologic examination, laryngoscopy, imaging, biopsy, and consideration of alternative or concurrent etiologies, is critical when patients present with nonstandard progression of disease.

## 4. Conclusion

This case highlights an unusual presentation of papillary thyroid carcinoma with suspected hypopharyngeal involvement in a patient with underlying PD, manifesting as progressive weakness and recurrent falls. Although subacute weakness and falls may suggest an exacerbation of PD, clinicians should maintain a high suspicion for head and neck pathology in patients with a history of thyroid malignancy and new‐onset hoarseness and dysphagia. Comprehensive diagnostic workup, including a detailed clinical history and targeted evaluation with either CT imaging or flexible laryngoscopy, is critical to uncover the underlying etiology of dysphagia or dysphonia.

## Funding

The authors received no specific funding for this work.

## Consent

No written consent was obtained as there are no patient identifiable data included in the case report.

## Conflicts of Interest

The authors declare no conflicts of interest.

## Data Availability

Data sharing is not applicable to this article as no datasets were generated or analyzed during the current study.

## References

[1] Escalante D. , Hohman M. H. , Sanders O. et al., Hypopharyngeal Cancer, StatPearls, 2025, StatPearls Publishing, Treasure Island, FL, https://www.ncbi.nlm.nih.gov/books/NBK567720/. 33620797

[2] Mousavi S. E. , Ilaghi M. , Mirzazadeh Y. , Mosavi Jarrahi A. , and Nejadghaderi S. A. , Global Epidemiology and Socioeconomic Correlates of Hypopharyngeal Cancer in 2020 and Its Projection to 2040: Findings From GLOBOCAN 2020, Frontiers in Oncology. (2024) 14, 10.3389/fonc.2024.1398063. PMC11402725 39286014

[3] Wycliffe N. D. , Grover R. S. , Kim P. D. , and Simental A. , Hypopharyngeal Cancer, Topics in Magnetic Resonance Imaging: TMRI. (2007) 18, no. 4, 243–258, 10.1097/RMR.0b013e3181570c3f. 17893590

[4] Piazza C. , Paderno A. , Ravanelli M. , and Pessina C. , Clinical and Radiological Evaluation of Hypopharyngeal Carcinoma, Advances in Oto-Rhino-Laryngology. (2019) 83, 35–46, 10.1159/000492306. 30943514

[5] Tan H. Y. , Sanudin S. H. , Lum S. G. , and Wong E. H. C. , An Unusual First Presentation of Hypopharyngeal Carcinoma as Thyroid Abscess: Case Report of a Diagnostic Challenge, International Journal of Surgery Case Reports. (2021) 81, no. C, 10.1016/j.ijscr.2021.105723. PMC7957141 33713999

[6] Toraih E. A. , Hussein M. H. , Zerfaoui M. et al., Site-Specific Metastasis and Survival in Papillary Thyroid Cancer: The Importance of Brain and Multi-Organ Disease, Cancers. (2021) 13, no. 7, 10.3390/cancers13071625. PMC8037301 33915699

[7] Du B. , Wang S. , Liu Y. , Li Y. , and Li X. , Metastatic Papillary Thyroid Cancer Mimicking Hypopharyngeal Carcinoma, Clinical Nuclear Medicine. (2023) 48, no. 1, 90–91, 10.1097/RLU.0000000000004468. 36469069

[8] Batıoğlu-Karaaltın A. , Azizli E. , Ersözlü I. , Yiğit O. , and Cansız H. , Hypopharyngeal and Parapharyngeal Space Metastasis of Papillary Thyroid Carcinoma: A Case Report, Balkan Medical Journal. (June 2014) 31, no. 2, 177–179, 10.5152/balkanmedj.2014.13003. 25207192 PMC4115933

[9] Lee I. H. and Kim D. K. , Head and Neck Cancer: A Potential Risk Factor for Parkinson’s Disease?, Cancers. (2024) 16, no. 13, 10.3390/cancers16132486. PMC11240437 39001548

[10] Schrag A. , Hommel A. L. A. J. , Lorenzl S. et al., The Late Stage of Parkinson’s-Results of a Large Multinational Study on Motor and Non-Motor Complications, Parkinsonism & Related Disorders. (2020) 75, 91–96, 10.1016/j.parkreldis.2020.05.016. 32505085

[11] Mu L. , Sobotka S. , Chen J. et al., Alpha-Synuclein Pathology and Axonal Degeneration of the Peripheral Motor Nerves Innervating Pharyngeal Muscles in Parkinson Disease, Journal of Neuropathology and Experimental Neurology. (2013) 72, no. 2, 119–129, 10.1097/NEN.0b013e3182801cde. 23334595 PMC3552335

[12] Mu L. , Sobotka S. , Chen J. et al., Altered Pharyngeal Muscles in Parkinson Disease, Journal of Neuropathology and Experimental Neurology. (2012) 71, no. 6, 520–530, 10.1097/NEN.0b013e318258381b. 22588389 PMC3358551

[13] Taira K. , Fujiwara K. , Fukuhara T. , Koyama S. , Morisaki T. , and Takeuchi H. , Evaluation of the Pharynx and Upper Esophageal Sphincter Motility Using High-Resolution Pharyngeal Manometry for Parkinson’s Disease, Clinical Neurology and Neurosurgery. (2021) 201, 10.1016/j.clineuro.2020.106447. 33421742

[14] Curtis J. A. , Molfenter S. M. , and Troche M. S. , Pharyngeal Area Changes in Parkinson’s Disease and Its Effect on Swallowing Safety, Efficiency, and Kinematics, Dysphagia. (2020) 35, no. 2, 389–398, 10.1007/s00455-019-10052-7. 31446478 PMC7513198

[15] Chu E. C. and Wong A. Y. , Mitigating Gait Decline in a Woman With Parkinson’s Disease: A Case Report, Journal of Medical Cases. (2022) 13, no. 3, 140–144, 10.14740/jmc3856. 35356393 PMC8929208

[16] Shakya S. , Prevett J. , Hu X. , and Xiao R. , Characterization of Parkinson’s Disease Subtypes and Related Attributes, Frontiers in Neurology. (2022) 13, 10.3389/fneur.2022.810038. PMC9167933 35677337

